# Comprehensive analysis of the associations between clinical factors and outcomes by machine learning, using post marketing surveillance data of cabazitaxel in patients with castration-resistant prostate cancer

**DOI:** 10.1186/s12885-022-09509-0

**Published:** 2022-04-29

**Authors:** Hirotaka Kazama, Osamu Kawaguchi, Takeshi Seto, Kazuhiro Suzuki, Hideyasu Matsuyama, Nobuaki Matsubara, Yuki Tajima, Taro Fukao

**Affiliations:** 1 Sanofi Specialty Care Medical Oncology, 3-20-2 Nishi-Shinjuku, Shinjuku, Tokyo 163-1488 Japan; 2 Sanofi Research and Development, 3-20-2 Nishi-Shinjuku, Shinjuku, Tokyo 163-1488 Japan; 3 Sanofi Medical Affairs, 3-20-2 Nishi-Shinjuku, Shinjuku, Tokyo 163-1488 Japan; 4 Department of Urology, Gunma University Graduate School of Medicine, 3-39-22 Showa-machi, Maebashi, Gunma 371-8511 Japan; 5 Department of Urology, Yamaguchi University Graduate School of Medicine, 1-1-1 Minami-Kogushi, Ube, Yamaguchi 755-8505 Japan; 6 Department of Medical Oncology, National Cancer Center Hospital East, 6-5-1 Kashiwanoha, Kashiwa, Chiba, 277-8577 Japan; 7 Sanofi Global Oncology, 450 Water Street, Cambridge, MA 02141 USA

**Keywords:** Cabazitaxel, Castration-resistant prostate cancer, Machine learning technology, Neutropenia, Outcome-associated clinical factors

## Abstract

**Background:**

We aimed to evaluate relationships between clinical outcomes and explanatory variables by network clustering analysis using data from a post marketing surveillance (PMS) study of castration-resistant prostate cancer (CRPC) patients.

**Methods:**

The PMS was a prospective, multicenter, observational study of patients with metastatic, docetaxel-refractory CRPC treated with cabazitaxel in Japan after its launch in 2014. Graphical Markov (GM) model-based simulations and network clustering in ‘R’ package were conducted to identify correlations between clinical factors and outcomes. Factors shown to be associated with overall survival (OS) in the machine learning analysis were confirmed according to the clinical outcomes observed in the PMS.

**Results:**

Among the 660 patients analyzed, median patient age was 70.0 years, and median OS and time-to-treatment failure (TTF) were 319 and 116 days, respectively. In GM-based simulations, factors associated with OS were liver metastases, performance status (PS), TTF, and neutropenia (threshold 0.05), and liver metastases, PS, and TTF (threshold 0.01). Factors associated with TTF were OS and relative dose intensity (threshold 0.05), and OS (threshold 0.01). In network clustering in ‘R’ package, factors associated with OS were number of treatment cycles, discontinuation due to disease progression, and TTF (threshold 0.05), and liver and lung metastases, PS, discontinuation due to adverse events, and febrile neutropenia (threshold 0.01). Kaplan–Meier analysis of patient subgroups demonstrated that visceral metastases and poor PS at baseline were associated with worse OS, while neutropenia or febrile neutropenia and higher number of cabazitaxel cycles were associated with better OS.

**Conclusions:**

Neutropenia may be a predictive factor for treatment efficacy in terms of survival. Poor PS and distant metastases to the liver and lungs were shown to be associated with worse outcomes, while factors related to treatment duration were shown to positively correlate with better OS.

**Supplementary Information:**

The online version contains supplementary material available at 10.1186/s12885-022-09509-0.

## Background

For patients with cancer, the identification and application of prognostic markers can assist in predicting clinical outcomes, facilitate treatment choice, and improve therapeutic research [1]. Various models can be used to predict the risk of the disease using data obtained from cohort studies or from randomized trials in multivariate analyses [2]. The use of machine learning algorithms is expected to improve the definitive identification of prognostic factors, due to its increased flexibility and enhanced performance compared with traditional statistical modeling techniques [3], although there remains room for improvement in machine learning methodology [4].

Castration-resistant prostate cancer (CRPC) is a form of prostate cancer that progresses despite the use of androgen depletion therapy. However, there are limitations associated with the biomarkers currently available for CRPC, adding to the challenges faced by physicians when making prognostic and therapeutic decisions [5]. Patients with CRPC may present with few symptoms but have rising levels of serum prostate-specific antigen (PSA), or they may have multiple metastases and significant morbidity [6]. Between 10 and 20% of patients with prostate cancer develop CRPC within 5 years, and a pooled survival estimate suggested that patients with CRPC could expect to live for 14 months following diagnosis (range 9 to 30 months) [7].

The standard first-line chemotherapy treatment for CRPC recommended by current guidelines includes systemic docetaxel plus concurrent steroids [6, 8, 9]. Data from clinical trials demonstrated benefits for enzalutamide (a novel androgen receptor signal inhibitor) [10], abiraterone (an androgen synthetic inhibitor) [11], and cabazitaxel (a second-generation taxane) [12] following docetaxel resistance, and their use in CRPC is now becoming widespread. In Japan, cabazitaxel is approved for use in patients following docetaxel resistance [9].

Several studies have reported on the efficacy of cabazitaxel in cancer therapy, with improvements in survival in pretreated men with CRPC in clinical trials [12–14] and correspondingly good survival outcomes in real-world practice [15–18]. One of the most common Grade ≥ 3 adverse events (AEs) associated with the use of cabazitaxel is neutropenia or febrile neutropenia [12, 18, 19] and, interestingly, there appears to be a correlation between efficacy and rates of Grade ≥ 3 neutropenia [20, 21]. Other prognostic factors that have been reported to be associated with clinical outcomes in CRPC patients treated with cabazitaxel include site of metastasis [18, 22–24], Eastern Cooperative Oncology Group performance status (ECOG PS) [24, 25], number of cabazitaxel treatment cycles [23], prior treatment history [18, 26] and several laboratory measures [25, 27, 28].

We have previously reported the safety and effectiveness of cabazitaxel in a post marketing surveillance (PMS) of 660 patients with CRPC [15–17]. An analysis of outcomes according to cabazitaxel dose suggested that a higher dose may extend overall survival (OS) and time-to-treatment failure (TTF), but it can also induce more events of neutropenia and febrile neutropenia [17]. It is unclear whether the dose of cabazitaxel or the development of neutropenia is the key to predicting survival outcomes. We conducted a network analysis of the data from the PMS to comprehensively evaluate the relationship between clinical factors and patient outcomes using machine learning technology. The objective of this exploratory signal-finding study was to identify correlations between clinical outcomes (response variables) and potential explanatory variables using a network clustering analysis.

## Methods

### Design of the PMS

The PMS was a prospective, multicenter, observational study, which registered all patients with metastatic, docetaxel-refractory CRPC treated with cabazitaxel following its launch in Japan in September 2014 [15]. The PMS was conducted in compliance with the Ministerial Ordinance on Good Post-marketing Study Practice for Drugs in Japan, was in line with Japanese law, and did not require patient consent for participation in accordance with local regulations and because data were collected anonymously.

Full details of the PMS have been reported [15]. In brief, a total of 660 patients were enrolled across 316 centers by June 2016. In general, cabazitaxel (25 mg/m^2^) was infused over 1 h every 3 weeks in combination with daily oral prednisolone, in accordance with the approved package insert [29]. Prophylactic granulocyte colony-stimulating factor was recommended for patients susceptible to febrile neutropenia.

Efficacy was assessed in terms of OS, evaluated from the date of first cabazitaxel administration to date of death from any cause; TTF, defined as the duration of cabazitaxel treatment; and PSA response rate, defined as a decrease of ≥ 30% from baseline where the PSA at baseline was ≥ 5 ng/mL.

Adverse drug reactions were evaluated according to Common Terminology Criteria for Adverse Events version 4.0. Effectiveness endpoints (OS, TTF, and PSA response) were assessed for up to 1 year.

### Machine learning analysis

Two types of analyses were conducted to identify correlations between patient and disease factors and clinical outcomes. For the first analysis, we conducted graphical Markov (GM) model-based simulations [30, 31] based on the partial correlation coefficient between response and explanatory variables. Response variables included OS, TTF, and PSA response. The 91 explanatory variables were derived from patient demographic and clinical characteristics at baseline, medical and treatment histories, and the AEs collected during the PMS. The choice of factors was based on the bootstrap method and *P*-value of each variable.

Response and explanatory variables are shown in Additional File 1. Simulations were performed in 1000 iterations, and the variables were considered as correlated when the *P*-value for the partial correlation coefficient was smaller than the defined alpha level (0.05 or 0.01) in more than 500 iterations. A graphical mixed model using the path consistency algorithm [32] was implemented to avoid interruption of analysis owing to the inability to calculate partial correlation coefficients between the variables. The statistical analysis and machine learning analysis were performed using ‘R’ (version 3.5.1) [33] to analyze and visualize the associations.

For the second analysis, we used network clustering in ‘R’ package (‘R’ version 3.5.1) to define the factors that were associated with the response variable(s) in more than four out of seven clustering models. The clustering models were edge betweenness, eigenvectors of matrices, community structure, fast unfolding of communities, near linear time algorithm, random walks, and maps of random walks [34]. The causality of association between variables was judged to be positive if the frequency of association was positive in 80% of the testing or if explanatory variables were repeatedly clustered into the same group as response variables.

### OS analysis by patient subgroup

Factors that were shown by the machine learning analysis to be associated with OS were confirmed according to the clinical outcomes observed in the PMS. Kaplan–Meier methodology was used to visualize the data; calculations were conducted using SAS software version 9.2 or 9.4 (SAS Institute Inc., Cary, NC, USA).

## Results

### Patients

Patient characteristics and cabazitaxel dosing conditions are shown in Table 1. The median patient age was 70.0 years, 97.9% had previously received docetaxel, 86.5% had received prior androgen receptor inhibitors, and 29.9% had received palliative radiation therapy. A total of 516 patients (78.2%) had a Gleason score of 8–10, and a range of metastatic sites were reported, including bone (88.0%), liver (13.3%), and lung (10.6%). The median number of cycles of cabazitaxel was 4.0 (min–max 1–18), and the median relative dose intensity (RDI) was 67.2% (min–max 17.8–101.0).

**Table 1:** Patient characteristics and cabazitaxel dosing conditions

	**Analysis set (*N* = 660)**
Age (years), median (min–max)	70.0 (43–91)
Gleason score of poorly differentiated (8–10), n (%)	516 (78.2)
ECOG PS, n (%)	
0	412 (62.4)
1	194 (29.4)
≥ 2	53 (8.0)
NA	1 (0.2)
Prior endocrine therapy, n (%)	
Enzalutamide	527 (79.9)
Abiraterone	363 (55.0)
Prior radiation therapy, n (%)	197 (29.9)
Metastatic sites when cabazitaxel treatment was initiated, n (%)	
None	6 (0.9)
Bone	581 (88.0)
Prostate	466 (70.6)
Regional lymph node	266 (40.3)
Distant lymph node	183 (27.7)
Liver	88 (13.3)
Seminal vesicle	78 (11.8)
Lung	70 (10.6)
Bladder	65 (9.9)
Other	36 (5.5)
Dose (mg/m^2^/cycle)	
Mean (SD)	20.8 (3.4)
Median (min–max)	20.0 (10.0–26.3)
< 15, n (%)	15 (2.3)
15 to < 20, n (%)	117 (17.7)
20 to < 25, n (%)	327 (49.6)
≥ 25, n (%)	199 (30.2)
Relative dose intensity (%)	
Mean (SD)	68.0 (16.4)
Median (min–max)	67.2 (17.8–101.0)
Number of treatment cycles	
Mean (SD)	5.5 (4.1)
Median (min–max)	4.0 (1–18)
Reason for treatment discontinuation^a^, n (%)	
Adverse event	259 (42.8)
Primary disease progression	341 (56.4)
Other	262 (43.3)

^a^Multiple reasons allowed

*ECOG PS* Eastern Cooperative Oncology Group performance status, *NA* not applicable, *SD* standard deviation

Treatment effectiveness and safety outcomes are reported in Table 2. Median OS was 319 days (95% confidence interval: 293–361) and median TTF was 116 days (95% confidence interval: 108–135). Neutropenia-associated events occurred in 382 (57.9%) patients, and 325 (49.2%) patients experienced Grade ≥ 3 events. Febrile neutropenia occurred in 119 (18.0%) patients.

**Table 2:** Treatment effectiveness and safety outcomes

	**Analysis set (*N* = 660)**
Overall survival, n (%)	656 (99.4)
Median (min–max), days (95% CI)	319 (293–361)
Events / censored cases	334 (50.6) / 322 (48.8)
Censored cases with 1-year survival	216 (32.7)
Time-to-treatment failure, n (%)	660 (100.0)
Median (min–max), days (95% CI)	116 (108–135)
Events / censored cases	581 (88.0) / 79 (12.0)
Censored cases with 1-year treatment	79 (12.0)
Adverse events^a^, n (%)	
Diarrhea	66 (10.0)
Renal impairment	1 (0.2)
Severe infectious disease	30 (4.5)
Anemia	94 (14.2)
Peripheral neuropathy	10 (1.5)
Bone marrow suppression (due to impaired hematopoiesis)	461 (69.8)
Neutropenia-associated events	382 (57.9)
Grade ≥ 3 event^b^	325 (49.2)
Febrile neutropenia	119 (18.0)

^a^Priority survey items; ^b^neutropenia, febrile neutropenia, and neutrophil count decreased

*CI* confidence interval

### GM model-based simulations

Table 3 shows the results of graphical modeling. When the threshold for correlation was set at 0.05 (Additional File 2a and Additional File 3), factors found to be associated with OS included presence of liver metastases, PS, TTF, and neutropenia. At a threshold value of 0.01 (Additional File 2b and Additional File 4), liver metastases, PS, and TTF retained their association but neutropenia did not retain its association. Factors associated with TTF were OS and RDI (threshold 0.05), and OS (threshold 0.01).

**Table 3:** Results of graphical Markov modeling

Proportion of estimations in which relationship was present (in 1000 iterations)	Causality association, positive: frequency > 50%	
	**Alpha level 0.05**	**Alpha level 0.01**
OS-associated factors		
TTF	1	1
OS censoring	0.83	0.77
ECOG PS	0.52	1
Observation period	1	1
Liver metastasis	0.76	0.76
Neutropenia	0.52	-
TTF-associated factors		
OS	1	1
TTF censoring	1	1
Observation period	1	1
RDI	1	-

*ECOG PS* Eastern Cooperative Oncology Group performance status, *OS* overall survival, *RDI* relative dose intensity, *TTF* time-to-treatment failure

### Network clustering in ‘R’ package using GM model-based simulations

The results of the clustering analysis are shown in Table 4. At a threshold of 0.05 (Additional File 5), the number of treatment cycles, discontinuation due to disease progression, and TTF correlated with OS. In addition, at a threshold of 0.01 (Additional File 6), liver and lung metastases, PS, discontinuation due to AEs and other reasons, and febrile neutropenia were found to be correlated with OS.

**Table 4:** Results of clustering analysis

Factor	Number of appearances in clusters			
	**Alpha level 0.05**		**Alpha level 0.01**	
	**OS**	**TTF**	**OS**	**TTF**
Treatment cycles	5	7	7	7
Lung metastasis	-	-	7	7
Liver metastasis	-	-	7	7
Observation period	5	7	7	7
OS	-	5	-	7
TTF	5	-	7	-
TTF censoring	5	7	7	7
ECOG PS	-	-	6	6
Febrile neutropenia	-	-	4	4
OS censoring	-	-	4	4
Treatment discontinuation (AE)	-	-	4	4
Treatment discontinuation (other)	-	-	4	4
Treatment discontinuation (PD)	4	4	4	4

*AE* adverse event, *ECOG PS* Eastern Cooperative Oncology Group performance status, *OS* overall survival, *PD* progressive disease, *TTF* time-to-treatment failure

### OS analysis by patient subgroup

**Fig. 1:**
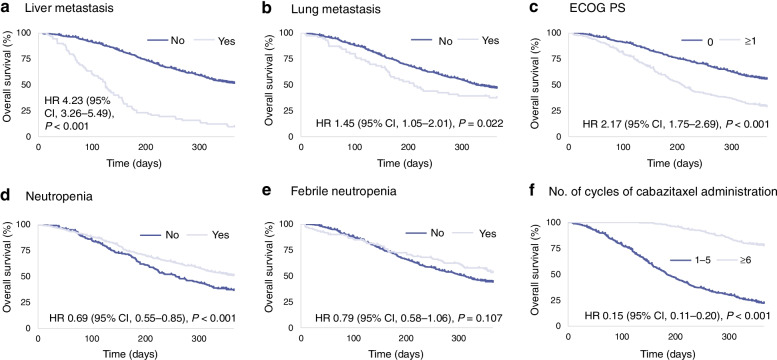
Kaplan–Meier analysis of OS for patient subgroups. **a:** liver metastasis; **b:** lung metastasis; **c:** ECOG PS; **d:** neutropenia; **e:** febrile neutropenia; **f:** number of cycles of cabazitaxel administration. *CI* confidence interval; *ECOG PS* Eastern Cooperative Oncology Group performance status; *HR* hazard ratio; *OS* overall survival

The results of the Kaplan–Meier analysis of OS for patient subgroups (Fig. 1) demonstrated that presence of visceral metastases and poor PS at baseline were associated with worse OS. Conversely, development of neutropenia or febrile neutropenia, and a higher number of cabazitaxel cycles were associated with better OS.

## Discussion

The definitive identification of prognostic factors for CRPC is critical for improving therapeutic decision-making. It is expected that the use of machine learning algorithms can overcome the limitations of conventional statistical assays, which have low levels of flexibility and performance restrictions due to the number of variables that can be evaluated [3]. Such algorithms can provide a much-needed new and robust information source to guide physicians in evaluating clinical risks and outcomes. This exploratory, signal-finding, machine learning analysis was intended to comprehensively identify factors associated with cabazitaxel in CRPC, using data from a PMS.

Previous analyses have suggested a positive correlation between cabazitaxel efficacy in terms of improved OS and/or progression-free survival and rates of cabazitaxel-induced Grade ≥ 3 neutropenia [20, 21]. These previous studies did not assess the relationship between cabazitaxel dosing and OS benefit. Data from our PMS suggested that a higher dose of cabazitaxel could extend OS but was also associated with an increased incidence of neutropenia [17]. The novelty of our study is that we further analyzed the PMS data to clarify whether the dose of cabazitaxel or the development of neutropenia was the key factor in predicting OS. Although neutropenia was found to be associated with OS, a relationship between the RDI of cabazitaxel and OS was not detected in this analysis, despite RDI in the network analysis being located near febrile neutropenia and neutropenia via TTF. The phase 3 PROSELICA study found that cabazitaxel 20 mg/m^2^ (reduced dose) was noninferior to cabazitaxel 25 mg/m^2^ (approved dose) in post-docetaxel patients with metastatic CRPC [14]. In that study, the reduced dose of cabazitaxel maintained ≥ 50% of the OS benefit of the approved dose versus mitoxantrone (data from the phase 3 TROPIC study), thus, the noninferiority endpoint was met. While fewer AEs were observed with the reduced dose, secondary endpoints, including progression-free survival and PSA, favored the approved dose.

In this analysis, factors associated with worse outcomes for men with metastatic CRPC included poor PS and presence of liver/lung metastases. This is in accordance with prior studies that have also identified PS [24, 25] and visceral metastases as indicators of poor prognosis [18, 22–24]. We consider that this concordance supports the results of this exploratory machine learning analysis.

Using the GM and clustering methodologies, respectively, neutropenia and febrile neutropenia were identified as factors correlated with clinical outcomes in our analyses. This confirms the significance of this AE in the treatment of CRPC with cabazitaxel, as previously reported [20, 21]. Development of neutropenia during various cancer treatments has also been linked with improved clinical outcomes for patients with other tumor types, suggesting that neutropenia could potentially be a predictive factor for cancer treatment efficacy [35–37].

Notably, at the thresholds set in this analysis, cabazitaxel dose and use of granulocyte colony-stimulating factor were not associated with OS. RDI was also not associated with OS. There was a weak and negative correlation between RDI and TTF, although it remains to be determined whether this could have indirectly influenced OS. Overall, these data suggest that neutropenia, rather than the dose-related parameters of cabazitaxel, is associated with OS.

The present study data also indicated correlations with OS for several factors related to the treatment period of cabazitaxel (including TTF, number of cycles, and discontinuation of treatment). Data from a recent retrospective analysis also suggested a link between the number of cabazitaxel treatment cycles and survival. Patients receiving ≥ 4 cycles had significantly longer OS than those who received < 4 cycles (*P* < 0.001) [24].

There is currently a great deal of interest in machine learning to predict clinical outcomes in oncology [38–40]. It is thought that the widespread use of this technique could revolutionize future oncologic management and assist in the implementation of precision medicine [41]. Although some technical refinements are still necessary, the evidential value of the data from this analysis is strengthened and supported by identifying several survival-associated factors detected in prior analyses. Other advantages of this machine learning methodology are the lack of limitation on included factors and inclusion of patients who undergo dose increases. As such, we consider that this methodology may be implemented to analyze a range of real-world data, including registry studies for oncology drugs, to provide physicians with critical information to assist with patient management.

The current analysis has several limitations, one of which is that the observation period of the PMS was limited to 1 year. In this study, there are two types of censored populations. One corresponds to the censored population within 365 days and the other to the population observed throughout the 365 days, after which observation was stopped. Both populations were labeled with the same variable, and the techniques used for handling missing outcomes and censored cases may have introduced bias into the results. In addition, no association between PSA response and outcome was observed in this analysis, possibly due to a partial lack of data and the difficulty of categorizing baseline PSA levels as parameters. A relationship between the dose of cabazitaxel and OS was not detected in the present study. This does not negate our previous findings that the initial cabazitaxel dose exerted an effect on the clinical outcomes; this may have been because we used a trimmed dataset for the calculation (the dataset of the previous analysis did not include patients who received an escalated dose of cabazitaxel from the initial dose), and because physicians tend to use the most appropriate dose for each patient. Additionally, the cabazitaxel dose is directly associated with the complications of infection or bone suppression [17], possibly because longer treatment increases the chance of a treatment-emergent AE, which would be a time-dependent factor. There may also have been limitations related to the explanatory parameters, as these sometimes represent a combination of clinically significant factors. For example, TTF may be due to an AE or progressive disease. Finally, it must be noted that this was an exploratory analysis, intended to identify signals, rather than a model validation study to prove prognostic relationships. We aimed to prove the value of machine learning technology in the assembly of a model that could be used to evaluate correlative associations; our study was not designed to prove causative pathophysiologic associations between patient or disease variables and the subsequent clinical outcomes. The correlation analysis was completed at model creation without further verification, and no definitive predictive value can be inferred. Our machine learning model did indicate several avenues of interest to be explored in terms of the relationships between variables and outcomes, and many of the results were consistent with those in the published literature. However, further studies will be required to validate such models with additional cohort data, and confirm or disprove hypotheses relating to prognostic ability and the attribution of causation.

## Conclusions

This analysis suggests that neutropenia may be correlated with treatment efficacy in terms of survival. Poor PS and distant metastases to the liver and lungs were determined to be associated with worse outcomes. In contrast, factors related to treatment duration were shown to positively correlate with improved OS. The identification of factors that have been previously reported to be associated with survival supports the results of our machine learning analysis and strengthens the value of this technique as a potentially powerful tool in the assessment and analysis of clinical risks and outcomes.

## Supplementary Information

**Additional file 1. **Response and explanatory variables. Table detailing the response and explanatory variables for the present study.

**Additional file 2.** Graphical model for partial correlation dependencies. **a** threshold 0.05; **b** threshold 0.01. Two part figure illustrating the partial correlation dependencies.

**Additional file 3.** Analysis results of graphical model (threshold 0.05). Data showing the graphical model analysis using a threshold of 0.05.

**Additional file 4.** Analysis results of graphical model (threshold 0.01). Data showing the graphical model analysis using a threshold of 0.01.

**Additional file 5.** Clustering analysis (threshold 0.05). Data showing the clustering analysis using a threshold of 0.05.

**Additional file 6.** Clustering analysis (threshold 0.01). Data showing the clustering analysis using a threshold of 0.01.

**Additional file 7.** List of institutions that provided permission to access the data. The names of institutions that provided permission to access the data for analysis.

**Additional file 8.**

## Data Availability

The datasets generated and/or analyzed during the current study are closed to public access, and links are not available. The use of each dataset is limited to the approved use from each participating institution, but datasets are available from the corresponding author on reasonable request. The authors have received administrative permission from each institution to access and use these data, and the names of institutions that provided permission to access the data are provided in Additional File 7.

## Abbreviations

AE: Adverse event
CRPC: Castration-resistant prostate cancer
ECOG PS: Eastern Cooperative Oncology Group performance status
GM: Graphical Markov
OS: Overall survival
PMS: Post-marketing surveillance
PSA: Prostate-specific antigen
RDI: Relative dose intensity
TTF: Time-to-treatment failure

## References

[1] Riley RD, Sauerbrei W, Altman DG (2009). Prognostic markers in cancer: the evolution of evidence from single studies to meta-analysis, and beyond. Br J Cancer.

[2] Moons KGM, Royston P, Vergouwe Y, Grobbee DE, Altman DG (2009). Prognosis and prognostic research: what, why, and how?. BMJ.

[3] Shimizu H, Nakayama KI (2020). Artificial intelligence in oncology. Cancer Sci.

[4] Christodoulou E, Ma J, Collins GS, Steyerberg EW, Verbakel JY, Van Calster B (2019). A systematic review shows no performance benefit of machine learning over logistic regression for clinical prediction models. J Clin Epidemiol.

[5] Boegemann M, Schrader AJ, Krabbe LM, Herrmann E (2015). Present, emerging and possible future biomarkers in castration resistant prostate cancer (CRPC). Curr Cancer Drug Targets.

[6] Saad F, Hotte SJ (2010). Guidelines for the management of castrate-resistant prostate cancer. Can Urol Assoc J.

[7] Kirby M, Hirst C, Crawford ED (2011). Characterising the castration-resistant prostate cancer population: a systematic review. Int J Clin Pract.

[8] National Comprehensive Cancer Network Clinical Practice Guidelines in Oncology: Prostate Cancer. Version 2.2021; February 17, 2021. https://www.nccn.org/professionals/physician_gls/pdf/prostate.pdf. Accessed 21 Dec 2020.

[9] Kakehi Y, Sugimoto M, Taoka R (2017). Evidenced-based clinical practice guideline for prostate cancer (summary: Japanese Urological Association, 2016 edition). Int J Urol.

[10] Scher HI, Fizazi K, Saad F, Taplin M-E, Sternberg CN, Miller K (2012). Increased survival with enzalutamide in prostate cancer after chemotherapy. N Engl J Med.

[11] de Bono JS, Logothetis CJ, Molina A, Fizazi K, North S, Chu L (2011). Abiraterone and increased survival in metastatic prostate cancer. N Engl J Med.

[12] de Bono JS, Oudard S, Ozgüroglu M, Hansen S, Machiels J-P, Kocak I (2010). Prednisone plus cabazitaxel or mitoxantrone for metastatic castration-resistant prostate cancer progressing after docetaxel treatment: a randomised open-label trial. Lancet.

[13] Bahl A, Oudard S, Tombal B, Ozgüroglu M, Hansen S, Kocak I (2013). Impact of cabazitaxel on 2-year survival and palliation of tumour-related pain in men with metastatic castration-resistant prostate cancer treated in the TROPIC trial. Ann Oncol.

[14] Eisenberger M, Hardy-Bessard AC, Kim CS, Géczi L, Ford D, Mourey L (2017). Phase III study comparing a reduced dose of cabazitaxel (20 mg/m^2^) and the currently approved dose (25 mg/m^2^) in postdocetaxel patients with metastatic castration-resistant prostate cancer-PROSELICA. J Clin Oncol.

[15] Suzuki K, Matsubara N, Kazama H, Seto T, Tsukube S, Matsuyama H (2019). Safety and efficacy of cabazitaxel in 660 patients with metastatic castration-resistant prostate cancer in real-world settings: results of a Japanese post-marketing surveillance study. Jpn J Clin Oncol.

[16] Matsubara N, Suzuki K, Kazama H, Tsukube S, Seto T, Matsuyama H (2020). Cabazitaxel in patients aged ≥80 years with castration-resistant prostate cancer: results of a post-marketing surveillance study in Japan. J Geriatr Oncol.

[17] Matsuyama H, Matsubara N, Kazama H, Seto T, Tsukube S, Suzuki K (2020). Real-world efficacy and safety of two doses of cabazitaxel (20 or 25 mg/m^2^) in patients with castration-resistant prostate cancer: results of a Japanese post-marketing surveillance study. BMC Cancer.

[18] Rouyer M, Oudard S, Joly F, Fizazi K, Tubach F, Jove J (2019). Overall and progression-free survival with cabazitaxel in metastatic castration-resistant prostate cancer in routine clinical practice: the FUJI cohort. Br J Cancer.

[19] Malik Z, Heidenreich A, Bracarda S, Ardavanis A, Parente P, Scholz H-J (2019). Real-world experience with cabazitaxel in patients with metastatic castration-resistant prostate cancer: a final, pooled analysis of the compassionate use prog. Oncotarget..

[20] Meisel A, von Felten S, Vogt DR, Liewen H, de Wit R, de Bono J (2016). Severe neutropenia during cabazitaxel treatment is associated with survival benefit in men with metastatic castration-resistant prostate cancer (mCRPC): a post-hoc analysis of the TROPIC phase III trial. Eur J Cancer.

[21] Kosaka T, Shinojima T, Morita S, Oya M (2018). Prognostic significance of grade 3/4 neutropenia in Japanese prostate cancer patients treated with cabazitaxel. Cancer Sci.

[22] Halabi S, Kelly WK, Ma H, Zhou H, Solomon NC, Fizazi K (2016). Meta-analysis evaluating the impact of site of metastasis on overall survival in men with castration-resistant prostate cancer. J Clin Oncol.

[23] Takai M, Kato S, Nakano M, Fujimoto S, Iinuma K, Ishida T (2021). Efficacy of cabazitaxel and the influence of clinical factors on the overall survival of patients with castration-resistant prostate cancer: a local experience of a multicenter retrospective study. Asia Pac J Clin Oncol.

[24] Halabi S, Lin CY, Kelly WK, Fizazi KS, Moul JW, Kaplan EB (2014). Updated prognostic model for predicting overall survival in first-line chemotherapy for patients with metastatic castration-resistant prostate cancer. J Clin Oncol.

[25] Belderbos BPS, de Wit R, Hoop EO, Nieuweboer A, Hamberg P, van Alphen RJ (2017). Prognostic factors in men with metastatic castration-resistant prostate cancer treated with cabazitaxel. Oncotarget..

[26] Yasuoka S, Yuasa T, Ogawa M, Komai Y, Numao N, Yamamoto S (2019). Risk factors for poor survival in metastatic castration-resistant prostate cancer treated with cabazitaxel in Japan. Anticancer Res..

[27] Yokom DW, Stewart J, Alimohamed NS, Winquist E, Berry S, Hubay S (2018). Prognostic and predictive clinical factors in patients with metastatic castration-resistant prostate cancer treated with cabazitaxel. Can Urol Assoc J.

[28] Uemura K, Kawahara T, Yamashita D, Jikuya R, Abe K, Tatenuma T (2017). Neutrophil-to-lymphocyte ratio predicts prognosis in castration-resistant prostate cancer patients who received cabazitaxel chemotherapy. Biomed Res Int.

[29] JEVTANA^®^ (cabazitaxel) package insert. https://pins.japic.or.jp/pdf/newPINS/00063089.pdf Accessed 21 Dec 2020.

[30] Whittaker J (1990). Graphical Models in Applied Multivariate Statistics.

[31] Pearl J. Causality. Cambridge: Cambridge University Press; 2009. 10.1017/CBO9780511803161.

[32] Saito S, Zhou X, Bae T, Kim S, Horimoto K (2013). Identification of master regulator candidates in conjunction with network screening and inference. Int J Data Min Bioinform.

[33] The R project for statistical computing. https://www.r-project.org/. Accessed 21 Dec 2020.

[34] igraph: network analysis and visualization. https://cran.r-project.org/web/packages/igraph/index.html. Accessed 21 Dec 2020.

[35] Kasi PM, Grothey A (2018). Chemotherapy-induced neutropenia as a prognostic and predictive marker of outcomes in solid-tumor patients. Drugs.

[36] Lalami Y, Klastersky J (2017). Impact of chemotherapy-induced neutropenia (CIN) and febrile neutropenia (FN) on cancer treatment outcomes: an overview about well-established and recently emerging clinical data. Crit Rev Oncol Hematol.

[37] McAndrew NP, Dickson MA, Clark AS, Troxel AB, O'Hara MH, Christopher C (2020). Early treatment-related neutropenia predicts response to palbociclib. Br J Cancer.

[38] Ferroni P, Zanzotto FM, Riondino S, Scarpato N, Guadagni F, Roselli M (2019). Breast cancer prognosis using a machine learning approach. Cancers (Basel).

[39] Akcay M, Etiz D, Celik O, Ozen A (2020). Evaluation of prognosis in nasopharyngeal cancer using machine learning. Technol Cancer Res Treat.

[40] Pan L, Liu G, Lin F, Zhong S, Xia H, Sun X (2017). Machine learning applications for prediction of relapse in childhood acute lymphoblastic leukemia. Sci Rep.

[41] Cuocolo R, Caruso M, Perillo T, Ugga L, Petretta M (2020). Machine learning in oncology: a clinical appraisal. Cancer Lett.

