# Supplementary material for: Comprehensive analysis of the associations between clinical factors and outcomes by machine learning, using post marketing surveillance data of cabazitaxel in patients with castration-resistant prostate cancer
Source: BMC Cancer. 2022 Apr 29;22:470. doi: 10.1186/s12885-022-09509-0 (PMC9052565; doi:10.1186/s12885-022-09509-0)
Supplement: Supplementary file 1 — Additional file 1. Response and explanatory variables. Table detailing the response and explanatory variables for the present study. [file 12885_2022_9509_MOESM1_ESM.docx]

# Additional File 1

# Comprehensive analysis of the associations between clinical factors and outcomes by machine learning, using post marketing surveillance data of cabazitaxel in patients with castration-resistant prostate cancer

Kazama et al

**Additional File 1** Response and explanatory variables

| **Variable** | **Output** | **Value** |
| --- | --- | --- |
| Age | AGE | Number |
| Height | HGTN | Number |
| Weight | WGTN | Number |
| Body surface area | BSAN | Number |
| Gleason score | GLEASON | 1: 2–4 2:5–7 3: 8–10 99: No data |
| ECOG performance status | POFST | 1: 0 2: 1 3: 2 4: 3 5: 4 |
| TNM classification/M | TNMMA | 1: M0 2: M1 3: MX 4: No record |
| TNM classification/N | TNMN | 0: No data 1: N0 2: N1 3: NX 77: No report |
| TNM classification/T | TNMTA | 1: T1 2: T2 3: T3 4: T4 5: TX 6:No record |
| Alcohol hypersensitivity | ALCO | 0: Absent 2: Present |
| Concomitant disease/history of allergy | COMPALGUM | 1: Present 2: Absent |
| Complications/infectious disease | COMPINFUM | 1: Present 2: Absent |
| Complications/kidney injury | COMPKIDUM | 1: Present 2: Absent |
| Complications/liver injury | COMPLIVUM | 1: Present 2: Absent |
| Drugs that strongly inhibit CYP3A4 | COMPMYEUM | 1: Present 2: Absent |
| Concomitant drugs/prophylactic G-CSF formulation | CONGCSFUM | 1: Present 2: Absent |
| Concomitant drugs/prevention of infectious disease | CONINFUM | 1: Present 2: Absent |
| Metastasis/none | FCS1 | −1: Applicable 0: Not applicable |
| Metastasis/prostate | FCS2 | −1: Present 0: Absent |
| Metastasis/bone | FCS3 | −1: Present 0: Absent |
| Metastasis/seminal vesicle | FCS4 | −1: Present 0: Absent |
| Metastasis/urinary bladder | FCS5 | −1: Present 0: Absent |
| Metastasis/lung | FCS6 | −1: Present 0: Absent |
| Metastasis/liver | FCS7 | −1: Present 0: Absent |
| Metastasis/regional lymph node | FCS8 | −1: Present 0: Absent |
| Metastasis/distant lymph node | FCS9 | −1: Present 0: Absent |
| Metastasis/others | FCS10 | −1: Present 0: Absent |
| Overall survival | OS | Number |
| Overall survival (censoring) | OS_CSR | Number |
| Time-to-treatment failure | TTF | Number |
| Time-to-treatment failure (censoring) | TTF_CSR | Number |
| Treatment duration | OBSTERM | Number |
| Number of cycles of cabazitaxel administration | DOSECYC | Number |
| Increase from the initial dose | DOSESCAL | 1: Present 2: Absent |
| Relative dose intensity | RDI | Number |
| Cumulative dose | DOSES | Number |
| PSA value before administration was started | PREPSA_A | Number |
| PSA response | PSAEFF4 | 1: Effective 2: Not effective |
| PSA time to progression | TPP4 | Number |
| PSA time to progression | PSAPRGTERM4 | Number |
| Cabazitaxel initial dose (cycle 1) | FSTDOSEN | 1: 15–<20 2: 20–<25 3: 25– |
| Prophylactic G-CSF in cycle 1 | FSTGCSFUM | 1: Present 2: Absent |
| Neutrophil count at baseline | INNEUN | Number |
| Myelosuppression (adverse drug reaction) | MYELOUM | 1: Present 2: Absent |
| Neutropenia (adverse drug reaction) | NEUTUM | 1: Present 2: Absent |
| Febrile neutropenia (FN) | FN | 0: Absent 1: Present |
| Grade ≥3 neutropenia | FN_2 | 0: Absent 1: Present |
| Diarrhea | AE_PCV_1 | 0: Absent 1: Present |
| Renal failure | AE_PCV_2 | 0: Absent 1: Present |
| Serious infectious disease | AE_PCV_3 | 0: Absent 1: Present |
| Anemia | AE_PCV_4 | 0: Absent 1: Present |
| Peripheral neuropathy | AE_PCV_5 | 0: Absent 1: Present |
| Discontinuation/development of adverse events (AEs) | STOP_AE | 0: No 1: Discontinued |
| Discontinuation/progress of the primary disease | STOP_PRS | 0: No 1: Discontinued |
| Discontinuation/others | STOP_OTH | 0: No 1: Discontinued |
| Treatment history/chemotherapy (other than docetaxel)/concomitant drug (estramustine) | PCHECE | 0: Absent 1: Present |
| Treatment history/chemotherapy (other than docetaxel)/concomitant drug (others) | PCHECO | 0: Absent 1: Present |
| Treatment history/chemotherapy (docetaxel)/the number of doses (75 mg) | PCHEDC1 | 1: <5 2: ≥5 |
| Treatment history/chemotherapy (docetaxel)/the number of doses (70 mg) | PCHEDC2 | 1: <5 2: ≥5 |
| Treatment history/chemotherapy (docetaxel)/the number of doses (60 mg) | PCHEDC3 | 1: <5 2: ≥5 |
| Treatment history/chemotherapy (docetaxel)/the number of doses (others) | PCHEDC4 | 1: <5 2: ≥5 |
| Treatment history/chemotherapy (docetaxel)/the number of doses | PCHEDCN | Number |
| Treatment history/chemotherapy (docetaxel)/duration | PCHEDDAY | Number |
| Treatment history/chemotherapy (docetaxel)/reason for discontinuation | PCHEDRSN | 0: NA 1: PD 2: AE |
| Treatment history/chemotherapy (docetaxel) | PCHEDUM | 1: Present 2: Absent |
| Treatment history/chemotherapy (other than docetaxel)/previous treatment (estramustine) | PCHEPE | 1: Present |
| Treatment history/chemotherapy (other than docetaxel)/previous treatment (others) | PCHEPO | 1: Present |
| Treatment history/chemotherapy (other than docetaxel) | PCHEUM | 1: Present 2: Absent |
| Presence or absence of prednisolone | PDLUM | 1: Present 2: Absent |
| Treatment history and concomitant drugs/endocrine therapy (abiraterone acetate)/duration | PHORADAY | Number |
| Treatment history and concomitant drugs/endocrine therapy (abiraterone acetate) | PHORAUM | 1: Present 2: Absent |
| Treatment history and concomitant drugs/endocrine therapy (enzalutamide)/duration | PHOREDAY | Number |
| Treatment history and concomitant drugs/endocrine therapy (enzalutamide) | PHOREUM | 1: Present 2: Absent |
| Treatment history and concomitant drugs/endocrine therapy/orchiectomy | PHORF1 | 1: Present |
| Treatment history and concomitant drugs/endocrine therapy/LH-RH agonist | PHORF2 | 1: Present |
| Treatment history and concomitant drugs/endocrine therapy/LH-RH antagonist | PHORF3 | 1: Present |
| Treatment history and concomitant drugs/endocrine therapy/steroidal antiandrogen | PHORF4 | 1: Present |
| Treatment history and concomitant drugs/endocrine therapy/nonsteroidal antiandrogen | PHORF5 | 1: Present |
| Treatment history and concomitant drugs/endocrine therapy/estrogenic drugs | PHORF6 | 1: Present |
| Treatment history and concomitant drugs/endocrine therapy/adrenocorticosteroids | PHORF7 | 1: Present |
| Treatment history and concomitant drugs/endocrine therapy/others | PHORF8 | 1: Present |
| Treatment history and concomitant drugs/endocrine therapy | PHORUM | 1: Present 2: Absent |
| Presence or absence of premedication | PMUM | 1: Present 2: Absent |
| Treatment history/radical local therapy | POPEUM | 1: Present 2: Absent |
| Presence or absence of primary resistance (reason for discontinuation: extracted by PD) | PR | 0:　1: |
| Novel AR inhibitor (or) | PREAR1 | 1: Present |
| Novel AR inhibitor (and) | PREAR2 | 1: Present |
| Novel AR inhibitor (classification) | PREAR3 | 1: 1 drug 2: 2 drugs |
| Switch from docetaxel to this drug (treatment immediately before this drug) | PREDTX | 1: Doc 2: ASI 3: Other |
| Sequence of treatment | SEQ | 1: Doc>Cab 2: Doc>ASI>Cab 3: ASI>Doc>Cab　4: ASI>Doc>ASI>Cab 5: Others |
| Treatment history and concomitant drugs/palliative radiation therapy/combination (vertebra) | RADIC1 | 1: Present |
| Treatment history and concomitant drugs/palliative radiation therapy/combination (other) | RADIC2 | 1: Present |
| Treatment history and concomitant drugs/palliative radiation therapy/pretreatment (vertebra) | RADIP1 | 1: Present |
| Treatment history and concomitant drugs/palliative radiation therapy/pretreatment (other) | RADIP2 | 1: Present |
| Treatment history and concomitant drugs/palliative radiation therapy | RADIUM | 1: Present 2: Absent |

*AR* androgen receptor; *ASI* androgen synthesis inhibitor; *ECOG PS* Eastern Cooperative Oncology Group performance status; *G-CSF* granulocyte colony-stimulating factor; *LH-RH* luteinizing hormone-releasing hormone; *PD* progressive disease; *PSA* prostate-specific antigen.
