# Supplementary material for: Comprehensive analysis of the associations between clinical factors and outcomes by machine learning, using post marketing surveillance data of cabazitaxel in patients with castration-resistant prostate cancer
Source: BMC Cancer. 2022 Apr 29;22:470. doi: 10.1186/s12885-022-09509-0 (PMC9052565; doi:10.1186/s12885-022-09509-0)
Supplement: Supplementary file 2 — Additional file 2. Graphical model for partial correlation dependencies. a threshold 0.05; b threshold 0.01. Two part figure illustrating the partial correlation dependencies. [file 12885_2022_9509_MOESM2_ESM.docx]

# Additional File 2

# Comprehensive analysis of the associations between clinical factors and outcomes by machine learning, using post marketing surveillance data of cabazitaxel in patients with castration-resistant prostate cancer

Kazama et al

**Additional File 2** Graphical model for partial correlation dependencies. **a** threshold 0.05; **b** threshold 0.01

**a**


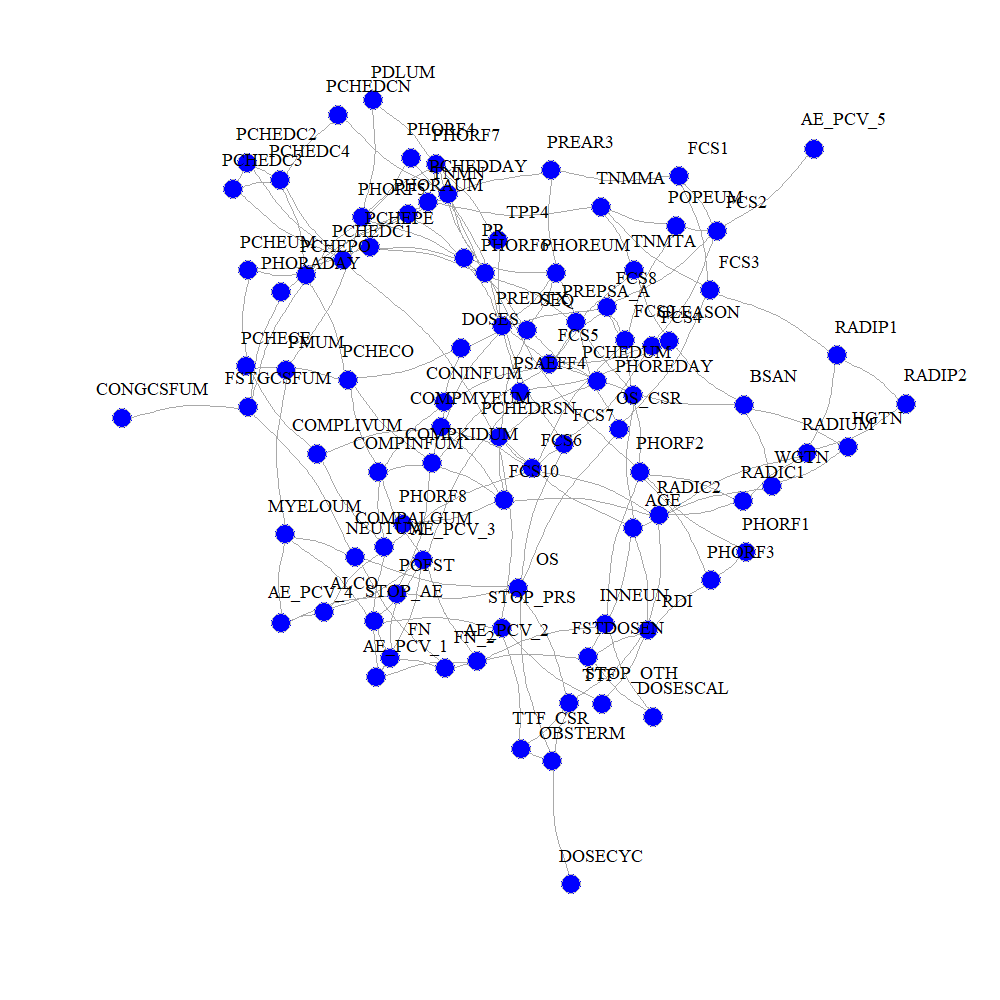


**b**

**
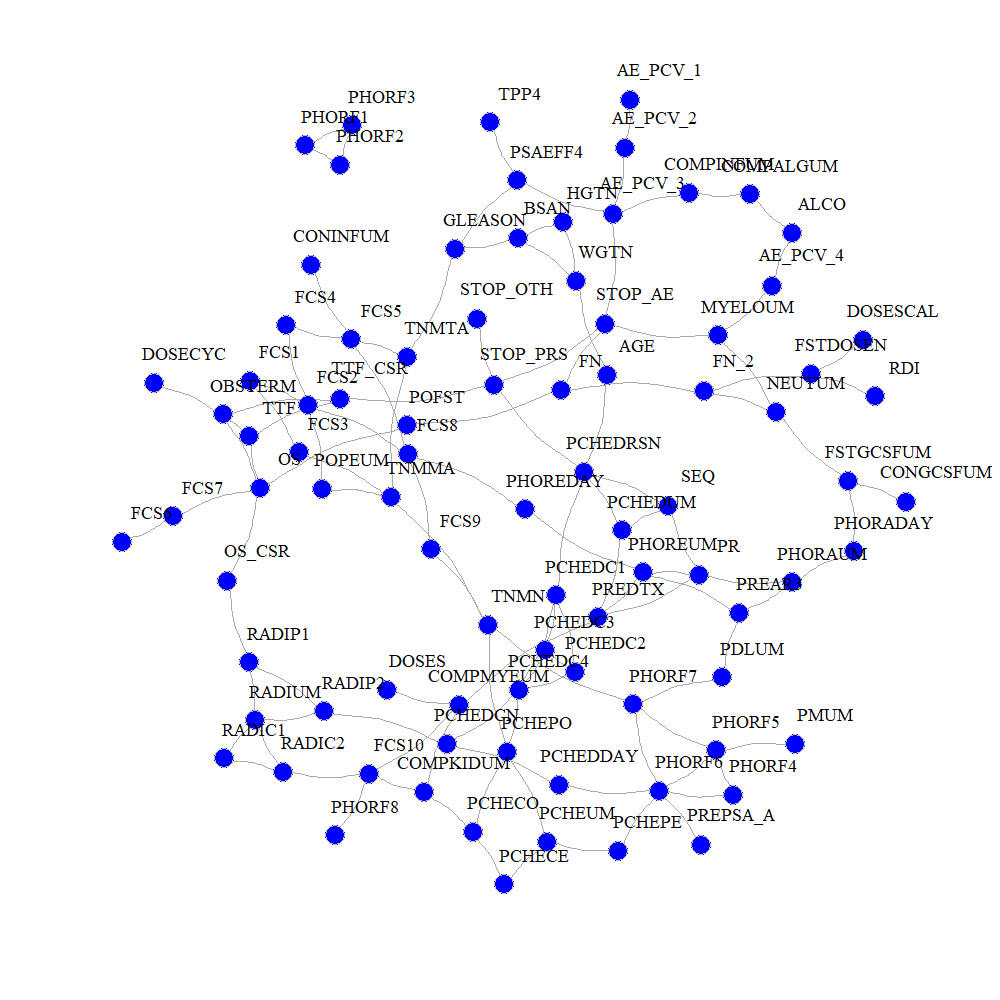
**
