# Supplementary material for: Comprehensive analysis of the associations between clinical factors and outcomes by machine learning, using post marketing surveillance data of cabazitaxel in patients with castration-resistant prostate cancer
Source: BMC Cancer. 2022 Apr 29;22:470. doi: 10.1186/s12885-022-09509-0 (PMC9052565; doi:10.1186/s12885-022-09509-0)
Supplement: Supplementary file 3 — Additional file 3. Analysis results of graphical model (threshold 0.05). Data showing the graphical model analysis using a threshold of 0.05. [file 12885_2022_9509_MOESM3_ESM.docx]

# Additional File 3

# Comprehensive analysis of the associations between clinical factors and outcomes by machine learning, using post marketing surveillance data of cabazitaxel in patients with castration-resistant prostate cancer

Kazama et al

**Additional File 3** Analysis results of graphical model (threshold 0.05)

**
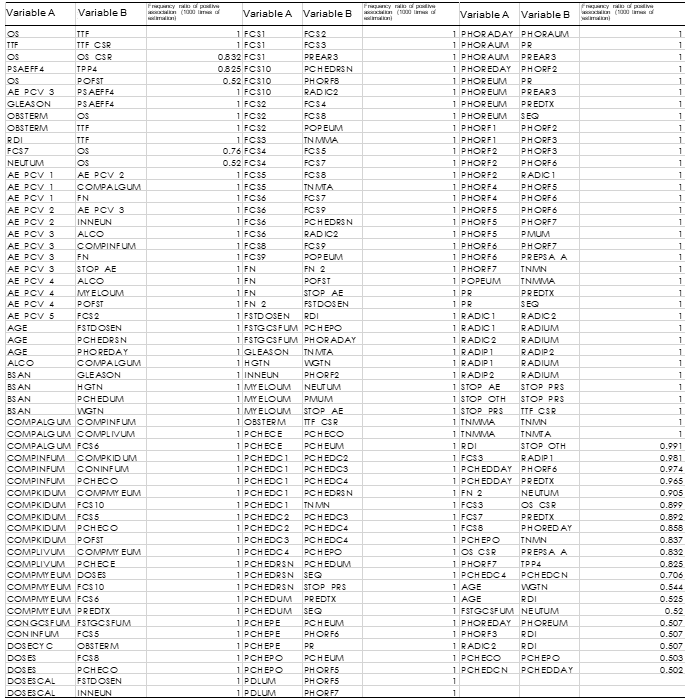
**
