# Supplementary material for: Comprehensive analysis of the associations between clinical factors and outcomes by machine learning, using post marketing surveillance data of cabazitaxel in patients with castration-resistant prostate cancer
Source: BMC Cancer. 2022 Apr 29;22:470. doi: 10.1186/s12885-022-09509-0 (PMC9052565; doi:10.1186/s12885-022-09509-0)
Supplement: Supplementary file 4 — Additional file 4. Analysis results of graphical model (threshold 0.01). Data showing the graphical model analysis using a threshold of 0.01. [file 12885_2022_9509_MOESM4_ESM.docx]

# Additional File 4

# Comprehensive analysis of the associations between clinical factors and outcomes by machine learning, using post marketing surveillance data of cabazitaxel in patients with castration-resistant prostate cancer

Kazama et al

**Additional File 4** Analysis results of graphical model (threshold 0.01)

**
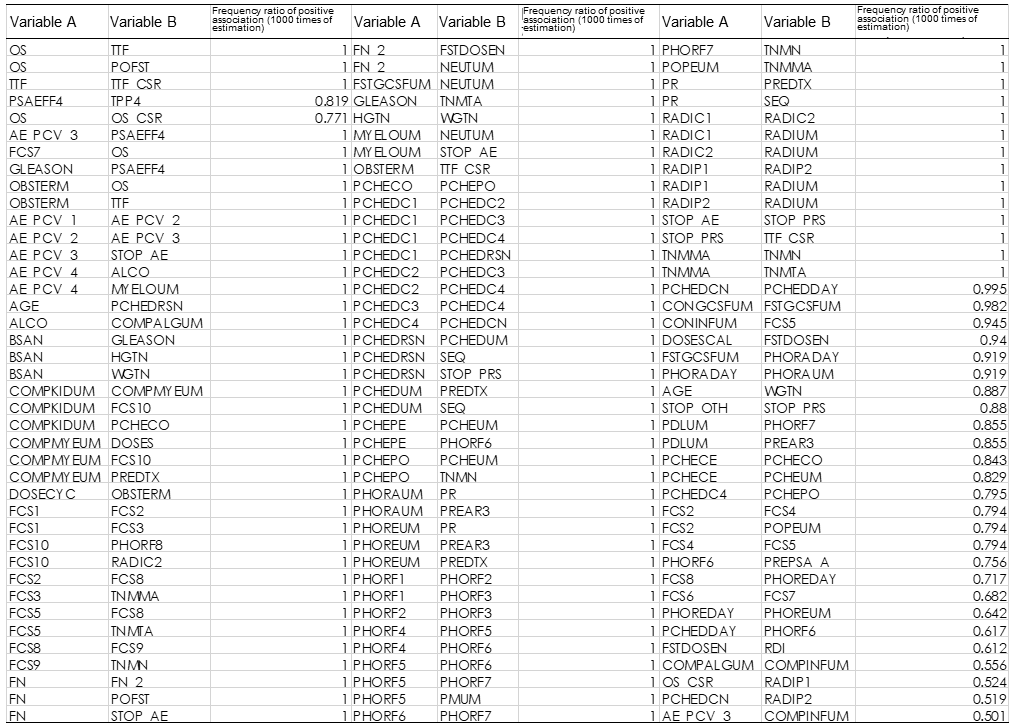
**
