# Supplementary material for: Comprehensive analysis of the associations between clinical factors and outcomes by machine learning, using post marketing surveillance data of cabazitaxel in patients with castration-resistant prostate cancer
Source: BMC Cancer. 2022 Apr 29;22:470. doi: 10.1186/s12885-022-09509-0 (PMC9052565; doi:10.1186/s12885-022-09509-0)
Supplement: Supplementary file 7 — Additional file 7. List of institutions that provided permission to access the data. The names of institutions that provided permission to access the data for analysis. [file 12885_2022_9509_MOESM7_ESM.docx]

# Additional File 7

# Comprehensive analysis of the associations between clinical factors and outcomes by machine learning, using post marketing surveillance data of cabazitaxel in patients with castration-resistant prostate cancer

Kazama et al

**Additional File 7** List of institutions that provided permission to access the data

Saka Urological Hospital

National Hospital Organization Hokkaido Cancer Center

Teine Keijinkai Hospital

Public Interest Association of Medical Service for Workers Kin-ikyo Chuo Hospital

Hakodate Municipal Hospital

Steel Memorial Muroran Hospital

Kitasaito Hospital

JCHO Sapporo Hokushin Hospital

Hakodate Goryoukaku Hospital

Hakodate General Central Hospital

Iwamizawa Municipal General Hospital

Asahikawa Medical University Hospital

Hirosaki University Hospital

Hachinohe City Hospital

Aomori City Hospital

Japanese Red Cross Morioka Hospital

Iwate Prefectural Central Hospital

Oshu City Mizusawa Hospital

Iwate Medical University Hospital

Senseki Hospital

Kesennuma City Hospital

Tohoku University Hospital

Sendai Medical Center

JCHO Sendai Hospital

Japanese Red Cross Ishinomaki Hospital

Japanese Red Cross Akita Hospital

Ogachi Central Hospital

Akita University Hospital

Akita City Hospital

Omagari Kosei Medical Center

Okitama Public General Hospital

Nihonkai General Hospital

Yamagata Prefectural Central Hospital

Yamagata University Hospital

Shirakawa Kosei General Hospital

Hoshi General Hospital

Takeda General Hospital

Ohara General Hospital

Southern Tohoku General Hospital

Ryugasaki Saiseikai Hospital

Tsukuba Medical Center Hospital

Mito Medical Center

Tokyo Medical University Ibaraki Medical Center

Ibaraki Prefectural Central Hospital

Mito Saiseikai General Hospital

Japanese Red Cross Mito Hospital

University of Tsukuba Hospital

Jichi Medical University Hospital

Saiseikai Utsunomiya Hospital

Sano City Hospital

International University of Health and Welfare Hospital

Gunma Prefectural Cancer Center

Tatebayashi Kosei General Hospital

Isesaki Municipal Hospital

Gunma University Hospital

Tone Chuo Hospital

Fujioka General Hospital

Dokkyo Medical University

Maruyama Memorial General Hospital

Saitama Medical University International Medical Center

Misato Central General Hospital

Saitama Ken-o Hospital

Sanaikai General Hospital

Higashimatsuyama Municipal Hospital

Soka Municipal Hospital

Kawaguchi Municipal Medical Center

Saitama Medical Center

Koshigawa Municipal Hospital

National Hospital Organization Saitama Hospital

Jichi Medical University Saitama Medical Center

Ageo Central General Hospital

National Cancer Center Hospital East

Chiba Aoba Municipal Hospital

Asahi General Hospital

Chiba Cancer Center

Japanese Red Cross Narita Hospital

Chiba Rosai Hospital

Toho University Medical Center Sakura Hospital

Mitsuwadai General Hospital

JCHO Funabashi Central Hospital

Center Hospital of the National Center for Global Health and Medicine

Juntendo Hospital

Tokyo Metropolitan Police Hospital

Tachikawa Hospital

Keio University Hospital

Takagi Hospital

National Cancer Center Hospital

Edogawa Hospital

Tokyo Medical And Dental University, Medical Hospital

Hino Municipal Hospital

Nihon University Itabashi Hospital

The Jikei University Hospital

National Hospital Organization Disaster Medical Center

Tama-Nambu Chiiki Hospital

Tobu Chiiki Hospital

Kyorin University Hospital

Tokyo Medical University Hachioji Medical Center

The University of Tokyo Hospital

Tokai University Hachioji Hospital

Cancer Institute Hospital

Tokyo Medical University Hospital

Tokyo Women's Medical University Hospital

Teikyo University Hospital

Fussa Hospital

Akiru Municipal Medical Center

Tokyo Women's Medical University Medical Center East

Tokyo Metropolitan Hiroo Hospital

Tokyo Metropolitan Cancer and Infectious Diseases Center Komagome Hospital

Toho University Medical Center Ohashi Hospital

Tokyo General Hospital

Japanese Red Cross Medical Center

Sagamidai Hospital

Hiratsuka City Hospital

Kitasato University Hospital

Sagamihara Kyodo Hospital

Yokohama City University Medical Center

Yokohama City University Hospital

Higashi-Totsuka Memorial Hospital

Hiratsuka Kyosai Hospital

Chigasaki Municipal Hospital

International Goodwill Hospital

Yokosuka Kyosai Hospital

Kanagawa Cancer Center

St. Marianna University School of Medicine Hospital

Kanto Rosai Hospital

Yokohama Rosai Hospital

Teikyo University Hospital, Mizonokuchi

Saiseikai Yokohamashi Tobu Hospital

Showa University Fujigaoka Hospital

Niigata Rinko Hospital

Asahi General Hospital

Saiseikai Toyama Hospital

Kouseiren Takaoka Hospital

Takaoka City Hospital

Tonami General Hospital

Kanazawa University Hospital

JCHO Kanazawa Hospital

Noto General Hospital

Public Central Hospital of Matto Ishikawa

Fukui Prefectural Hospital

University of Fukui Hospital

Fukui-ken Saiseikai Hospital

Fukui Red Cross Hospital

Yamanashi Prefectural Central Hospital

Nagano Municipal Hospital

Shinonoi General Hospital

Nagano Matsushiro General Hospital

Shinshu University Hospital

Kizawa Memorial Hospital

Ogaki Municipal Hospital

Gifu University Hospital

Chutoen General Medical Center

Hamamatsu University Hospital

Shizuoka General Hospital

Japanese Red Cross Shizuoka Hospital

National Hospital Organization Shizuoka Medical Center

Municipal Kosai Hospital

Seirei Mikatahara General Hospital

Shizuoka Saiseikai General Hospital

Yaizu City Hospital

Juntendo University Shizuoka Hospital

JA Shizuoka Kohseiren Enshu Hospital

Handa City Hospital

Okazaki City Hospital

Shinshiro Municipal Hospital

Fujita Health University Hospital

Komaki City Hospital

Aichi Medical University Hospital

Toyohashi Municipal Hospital

Japanese Red Cross Aichi Medical Center Nagoya Daiichi Hospital

Kariya Toyota General Hospital

JCHO Chukyo Hospital

Anjo Kosei Hospital

Nagoya University Hospital

Konan Kosei Hospital

Nagoya Memorial Hospital

Japanese Red Cross Nagoya Daini Hospital

Ichinomiyanishi Hospital

Matsusaka Chuo General Hospital

Mie Prefectural Shima Hospital

Hikone Municipal Hospital

Omihachiman Community Medical Center

Nagahama Red Cross Hospital

Nagahama City Hospital

Toyosato Hospital

Shiga University of Medical Science Hospital

Kyoto University Hospital

University Hospital Kyoto Prefectural University of Medicine

National Hospital Organization Kyoto Medical Center

Maizuru Kyosai Hospital

Ijinkai Takeda General Hospital

Kyoto Chubu Medical Center (previously Nantan General Hospital)

Japanese Red Cross Kyoto Daini Hospital

Kyoto Okamoto Memorial Hospital (previously Daini Okamoto General Hospital)

Kyoto Katsura Hospital

Takatsuki Red Cross Hospital

Rinku General Medical Center

Sakai City Medical Center

Ikeda City Hospital

Fuchu Hospital

Osaka City University Hospital

Kindai University Hospital

PL Hospital

Osaka University Hospital

Kansai Medical University Hospital (previously Kansai Medical University Hirakata Hospital)

Osaka Police Hospital

Medical Corporation Kyoujinkai Komatsu Hospital

Sakai Sakibana Hospital (previously Kindai University Sakai Hospital)

Osaka General Medical Center

Sumitomo Hospital

Daini Osaka Police Hospital (previously NTT West Osaka Hospital)

Noe Hospital

Yao Municipal Hospital

Osaka International Cancer Institute (previously Osaka Medical Center for Cancer and Cardiovascular Diseases)

Izumi City General Hospital (previously Izumi Municipal Hospital)

Kitano Hospital

Saiseikai Suita Hospital

Osaka Kaisei Hospital

Suita Municipal Hospital

Osaka Gyoumeikan Hospital

Osaka City General Hospital

Osaka Red Cross Hospital

Osaka Medical and Pharmaceutical University Hospital

JCHO Osaka Hospital

Kobe City Medical Center General Hospital

Hyogo Prefectural Amagasaki General Medical Center (previously Hyogo Prefectural Amagasaki Hospital)

Shinko Hospital

Honjo Kobe Research Center for Biomedical Innovation

Kobe University Hospital

Japanese Red Cross Society Himeji Hospital

Hyogo Prefectural Awaji Medical Center

Akashi City Hospital

Tsukazaki Hospital

Kobe City Nishi-Kobe Medical Center

Kobe City Medical Center West Hospital

Hyogo Prefectural Kakogawa Medical Center

Ako City Hospital

Nara Medical University Hospital

Yamato Takada Municipal Hospital

Nara Prefectural Seiwa Medical Center

Kindai University Nara Hospital

Wakayama Medical University Hospital

Japanese Red Cross Wakayama Medical Center

Tottori City Hospital

Japanese Red Cross Tottori Hospital

Tottori Prefectural Central Hospital

Tottori University Hospital

Shimane University Faculty of Medicine

Shimane Prefectural Central Hospital

Oda Municipal Hospital

General Medical Center Okayama City Municipal Hospital

Okayama University Hospital

Okamura Isshindo Hospital

Japanese Red Cross Mihara Hospital

Hiroshima University Hospital

Kure Kyosai Hospital

Federation of National Public Services and Affiliated Personnel Mutual Aid Associations Kure Kyosai Hospital

Hiroshima City Asa Citizens Hospital

JA Hiroshima General Hospital

Chugoku Rosai Hospital

Hiroshima City Hiroshima Citizens Hospital

Yamaguchi-ken Saiseikai Shimonoseki General Hospital

National Hospital Organization Kanmon Medical Center

Tsushimi Hospital

Yamaguchi University Hospital

Tokuyama Central Hospital

Shimonoseki City Toyoura Hospital

Tokushima Prefectural Welfare Agricultural Cooperative Association, Oe Kyodo Clinics

Yoshinogawa Medical Center

Tokushima University Hospital

Japanese Red Cross Tokushima Hospital

Tokushima Prefectural Central Hospital

Saint Martin's Hospital

Uchinomi hospital

Kagawa Rosai Hospital

National Hospital Organization Shikoku Cancer Center

Ehime University Hospital

Ehime Rosai Hospital

Sumitomo Besshi Hospital

Ehime Prefectural Central Hospital

Kochi Medical School Hospital

Hospital of the University of Occupational and Environmental Health, Japan

Tagawa Municipal Hospital

National Hospital Organization Kyushu Cancer Center

Kitakyushu General Hospital

Kyushu Rosai Hospital

Kokura Kinen Hospital

Saiseikai Yahata General Hospital

Fukuoka University Hospital

National Hospital Organization Kyushu Medical Center

Shin Yukuhashi Hospital

Kyushu University Hospital

Kurume University Hospital

Japanese Red Cross Fukuoka Hospital

Hamanomachi Hospital

Harasanshin Hospital

Yame General Hospital

Kano Hospital

Japan Community Health care Organization, Isahaya General Hospital

Nagasaki Goto Chuoh Hospital

Nagasaki Minato Medical Center Municipal Hospital (previously Nagasaki Harbor Medical Center City Hospital)

Japanese Red Cross Nagasaki Genbaku Hospital

Kumamoto Chuo Hospital

Kumamoto University Hospital

Japanese Red Cross Kumamoto Hospital

Japanese Red Cross Oita Hospital

Nakatsu Municipal Hospital

Oita Prefectural Hospital

National Hospital Organization Miyakonojo Medical Center (previously National Hospital Organization Miyakonojo Hospital)

Fujimoto Medical System Fujimoto General Hospital

Faculty of Medicine, University of Miyazaki Hospital

Medical Corporation Kakuyuukai Tsuruta Hospital

Kensho-Kai Hospital

Kimotsuki-gun Medical Association Hospital

Niimura Hospital

Kagoshima City Hospital

Saiseikai Sendai Hospital

University of the Ryukyus Hospital

Nanbu Tokushukai Hospital
