# Supplementary material for: Comprehensive analysis of the associations between clinical factors and outcomes by machine learning, using post marketing surveillance data of cabazitaxel in patients with castration-resistant prostate cancer
Source: BMC Cancer. 2022 Apr 29;22:470. doi: 10.1186/s12885-022-09509-0 (PMC9052565; doi:10.1186/s12885-022-09509-0)
Supplement: Supplementary file 8 — Additional file 8. [file 12885_2022_9509_MOESM8_ESM.pdf]

Predicting outcomes with machine learning: a study of patients with castration-resistant prostate cancer

March 2022

Study summary

Machine learning is a powerful tool that can help predict clinical outcomes in a range of diseases.

This study used machine learning to identify factors that could predict clinical outcomes in patients with castration-resistant prostate cancer being treated with cabazitaxel.

Better outcomes were associated with neutropenia and treatment duration.

Worse outcomes were associated with poor performance status and the presence of tumors in the liver or lungs.

Why was this study conducted?

A wide range of factors can contribute to outcomes of an illness or treatment. Identifying these factors, termed prognostic factors, can improve our understanding of a disease, and can contribute to the optimization of treatment strategies for an individual patient.

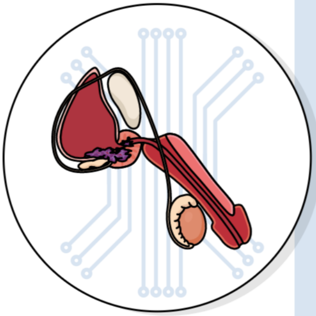

While statistical modelling is commonly used to identify prognostic factors, machine learning algorithms may lead to better identification of prognostic factors through increased flexibility and enhanced performance.

Previous studies have identified several factors that may predict clinical outcomes in patients with CRPC, including neutropenia. Higher doses of taxane, such as docetaxel and cabazitaxel, have been shown to improve survival, but may also lead to more cases of neutropenia. It is therefore unclear whether the dose of cabazitaxel or the development of neutropenia is the key to predicting survival.

What was the aim of the study?

The aim of this study was to identify the key factors that may predict clinical outcomes in patients with CRPC being treated with cabazitaxel using machine learning.

How was the study conducted?

This study used machine learning to analyze data from a previously published study of 660 adult male patients with CRPC who were previously treated with docetaxel. Two types of analyses, graphical Markov model-based simulations and network clustering in 'R' packages, were conducted to identify relationships between the following potential patient factors and clinical outcomes:

- **Patient factors:** These included patient demographics, clinical features at baseline, performance status (which describes a patient's level of functioning), medical and treatment histories, and side effects.

- **Clinical outcomes:** These included overall survival, the time-to-treatment failure, and prostate-specific antigen response rate (a common marker of treatment effect).

The factors that were identified by machine learning analysis to predict overall survival were then confirmed by comparison with the clinical outcomes observed in the original clinical trial.

Q&A

What is castration-resistant prostate cancer (CRPC)?

The disease progression of prostate cancer is typically slowed by lowering testosterone levels, as occurs in hormone therapy or castration. CRPC is a form of prostate cancer that does not respond to changes in hormones.

What is cabazitaxel?

Cabazitaxel is an anti-cancer chemotherapy drug that is approved for the treatment of CRPC in patients who are previously treated with docetaxel. Previous studies have demonstrated that it is effective and tolerable, and can improve survival in men with CRPC.

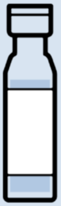

What is neutropenia?

Neutropenia is a condition in which patients have a low level of neutrophils, a type of white blood cell. It is one of the most common side effects that can occur from cabazitaxel treatment and makes patients susceptible to infections.

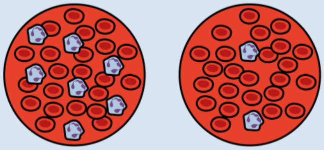

Normal blood cells

Neutropenia

What are the main results of the study?

Better clinical outcomes

Were associated with factors related to treatment duration.

The presence of neutropenia in CRPC patients treated with cabazitaxel may be a predictive factor in terms of overall survival

In contrast, no significant association between the dose-related parameters of cabazitaxel and treatment outcomes was shown.

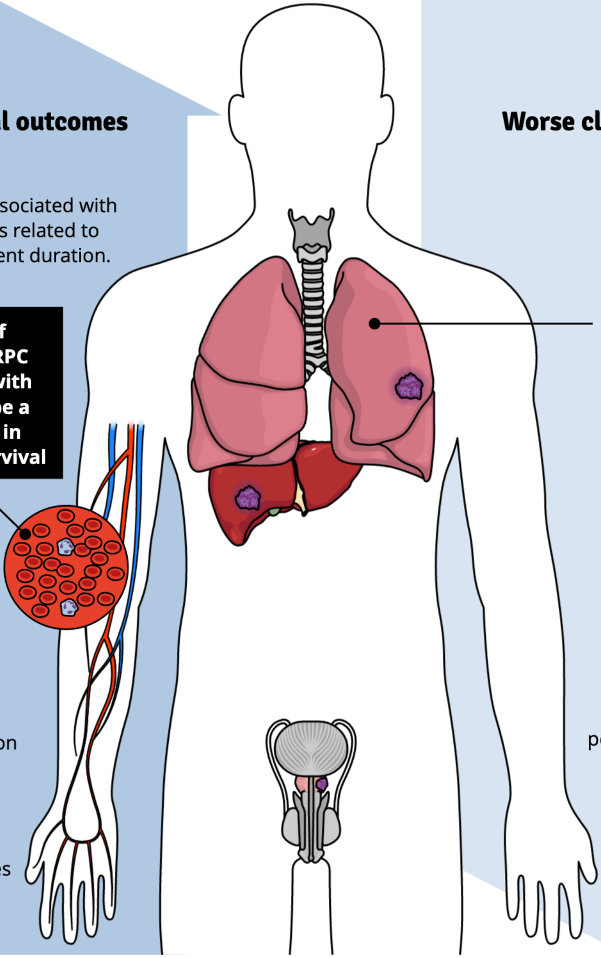

Worse clinical outcomes

Presence of tumors in the liver and lungs.

Related to poor performance status

Key takeaway

Machine learning analysis predicts association of neutropenia with improved overall survival in CRPC patients treated with cabazitaxel. Machine learning technology may be a useful tool in identifying factors associated with treatment outcomes.

Original publication: **Kazama H, Kawaguchi O, Seto T, Suzuki K, Matsuyama H, Matsubara N, Tajima Y, Fukao T. Comprehensive analysis of the associations between clinical factors and outcomes by machine learning, using post marketing surveillance data of cabazitaxel in patients with castration-resistant prostate cancer. BMC Cancer. 2022.**
Summary prepared by **Edanz.**
